# Water Transmission Increases the Intensity of COVID-19 Outbreaks

**DOI:** 10.3389/fpubh.2022.808523

**Published:** 2022-05-25

**Authors:** Jianping Huang, Xinbo Lian, Yingjie Zhao, Danfeng Wang, Siyu Chen, Li Zhang, Xiaoyue Liu, Jinfeng Gao, Chuwei Liu

**Affiliations:** Collaborative Innovation Center for West Ecological Safety (CIWES), College of Atmospheric Sciences, Lanzhou University, Lanzhou, China

**Keywords:** COVID-19, water transmission, ecological security, SEIR, natural disasters

## Abstract

India suffered from a devastating 2021 spring outbreak of coronavirus disease 2019 (COVID-19), surpassing any other outbreaks before. However, the reason for the acceleration of the outbreak in India is still unknown. We describe the statistical characteristics of infected patients from the first case in India to June 2021, and trace the causes of the two outbreaks in a complete way, combined with data on natural disasters, environmental pollution and population movements etc. We found that water-to-human transmission accelerates COVID-19 spreading. The transmission rate is 382% higher than the human-to-human transmission rate during the 2020 summer outbreak in India. When syndrome coronavirus 2 (SARS-CoV-2) enters the human body directly through the water-oral transmission pathway, virus particles and nitrogen salt in the water accelerate viral infection and mutation rates in the gastrointestinal tract. Based on the results of the attribution analysis, without the current effective interventions, India could have experienced a third outbreak during the monsoon season this year, which would have increased the severity of the disaster and led to a South Asian economic crisis.

## Introduction

Coronavirus disease 2019 (COVID-19) has inflicted great harm to human life worldwide and has posed an extreme threat in many countries (1). As the 2nd most populous country with a developing urban economy, India is still suffering disproportionately from COVID-19 (2). A high population density, health inequity, growing economic and social disparities, and unique cultural values pose challenges to the response to the epidemic by the Indian government (3). In the spring of 2021, India suffered a severe outbreak with the highest number of daily new cases in the world at the time, peaking at over 410,000. As of 1 June 2021, the cumulative number of confirmed cases reached 28,000,000. However, the reasons for such a rapid and unprecedented regional outbreak remain unclear.

The primary mechanisms of severe acute respiratory syndrome coronavirus 2 (SARS-CoV-2) transmission are respiratory droplet and contact transmission, including airborne transmission, fecal transmission, maternal-fetal transmission, etc, and it can survive for long periods outside of their host organism (4–7). Hospital air was shown to have SARS-CoV-2 levels of 9-219 COVID-19 viruses/m^3^, mediating long-range human-to-human transmission via air movement (8). SARS-CoV-2 can survive in stool samples for 1–2 days (9). Studies have also shown that the times for 90% reduction (T_90_) of viable SARS-CoV-2 in wastewater and tap water at room temperature were 1.5 and 1.7 days, respectively. In high-starting titer experiments, infectious virus persisted for the entire 7-day sampling time course (10). Contaminated water is likely to be a potential source of human exposure if aerosols are generated. The traditional disinfection method is expected to eradicate SARS-CoV-2 in sewage (11). However, overcrowded living conditions and poor sewage treatment practices in India may allow the virus to survive for prolonged periods of time (12), adding another potential transmission route.

To provide a basis for future global preparedness, we did a retrospective study on a huge data set of more than hundred million cases of COVID-19 worldwide. Attribution analysis of the dynamics data of cases was performed in India's two outbreaks. Combined with the simulation results, the triggers and mainly transmission routes of the epidemic in the 2021 spring severe outbreak in India was identified. We also simulated the development of epidemic situation without lockdown based on the findings, which served as an early warning.

## Materials and Methods

### Data Source

In this study, we used statistical dynamics and epidemiological modeling methods to identify the possible mechanisms of the outbreak. The results of attribution analysis are helpful for developing epidemic prevention plans to allow for the rational allocation of medical resources, especially in areas with more severe outbreaks. Data on global COVID-19 confirmed cases are from the COVID-19 Data Repository by the Center for Systems Science and Engineering (CSSE) at Johns Hopkins University (https://github.com/CSSEGISandData/COVID-19). Data sources for COVID-19 confirmed cases in the Indian states are given by: https://www.covid19india.org/. Data on rainfall in India's states and the number of flood victims come from the National Disaster Management Authority Government of India (https://ndmindia.mha.gov.in/reports#). The time, place and the coordinates of the bathing ghat of the Indian Kumbh Mela event in 2021 are given by: https://www.kumbhamela.net/. Water quality data of Ganges River in Uttar Pradesh were obtained from Uttar Pradesh Pollution Control Board (UPPCB): http://www.uppcb.com/river_quality.htm.

### EEMD Method

The ensemble empirical mode decomposition (EEMD) method was used to analyze the influence of the number of flooding victims on the number of COVID-19 cases. EEMD is a time series analysis method based on empirical mode decomposition (EMD) (13). EEMD decomposes complex data series into finite quasiperiodic components at different frequencies and is suitable for adaptive analysis of nonlinear and nonstationary time series. EMD/EEMD methods have been used to analyze and process nonlinear and nonstationary climate and ocean data, biomedical signals, financial signals, and COVID-19 simulation predictions. The decomposition process of the EEMD method can be shown as follows: First, white noise series *w(t)x(t)* is added, and then EMD method is used to decompose the new time series into Intrinsic Mode Functions (IMFs) terms. In the third step, different white noise series are used to repeat the first and second steps, and the results are added to the original time series each time. Finally, the set of IMF items in the EMD method is averaged (14).

### Model Simulation

Here, the second version of Global Prediction System for the COVID-19 Pandemic (GPCP) developed by Lanzhou University (http://covid-19.lzu.edu.cn/) was used for epidemiological simulations (15). The GPCP system was used to simulate and predict the trend of the epidemic in India, based on a modified version of the suspected, exposed, infectious, recovered (SEIR) epidemiological model. The theoretical framework is based on the division of the human host population into categories containing susceptible, infected but not yet infectious (exposed), infectious, and recovered individuals (16). These susceptible-exposed-infectious-recovered models are usually expressed as a system of differential equations.

The original model can be described by the following ordinary differential equations:

(1) $$\begin{array}{r} \frac{dS\left(t\right)}{dt}=-\frac{\beta I\left(t\right)S\left(t\right)}{N} \end{array}$$

(2) $$\begin{array}{r} \frac{dE\left(t\right)}{dt}=\frac{\beta I\left(t\right)S\left(t\right)}{N}-\gamma E\left(t\right) \end{array}$$

(3) $$\begin{array}{r} \frac{dI\left(t\right)}{dt}=\gamma E\left(t\right)-\left(\lambda +\kappa \right)I\left(t\right) \end{array}$$

(4) $$\begin{array}{r} \frac{dR\left(t\right)}{dt}=\left(\lambda +\kappa \right)I\left(t\right) \end{array}$$

The susceptible (S) refer to people who are not sick but lack immunity and are vulnerable to infection after coming into contact with the infected. In the absence of effective pharmaceutical treatments and vaccines, all populations are at risk of infection when they are exposed to the virus. The exposed (E) refer to people who have been in contact with an infected person but are not contagious. Such people can play a big role in the spread of infectious diseases with long incubation periods. They may also have the potential to spread the virus. The infective (I) represent the infective people with infectious capacity. The infective can spread virus to the susceptible, turning them into the exposed or the infective. The recovered (R) are those who recover or die of the disease. If it is a lifelong immune infectious disease, R cannot be changed into S, E or I again. If the immune period is limited, R can be changed into S again, and then be infected. In this paper, our modifications to the epidemiological model are based on the model of Peng et al. (17). The modified SEIR model defines seven states of disease: susceptible cases (S), protected cases (P), potentially infected cases (E, infected cases in a latent period), infected cases (I, infected cases that have not been quarantined), quarantined cases (Q, confirmed and quarantined cases), recovered cases (R), and cases of mortality (D). The sum of the seven categories is equal to the total population (N) at any time. The model contains the following equations:

(5) $$\begin{array}{r} \frac{dS\left(t\right)}{dt}=-\frac{\beta \left(t\right)I\left(t\right)S\left(t\right)}{N}-\alpha S\left(t\right) \end{array}$$

(6) $$\begin{array}{r} \frac{dP\left(t\right)}{dt}=\alpha S\left(t\right) \end{array}$$

(7) $$\begin{array}{r} \frac{dE\left(t\right)}{dt}=\frac{\beta \left(t\right)I\left(t\right)S\left(t\right)}{N}-\gamma E\left(t\right) \end{array}$$

(8) $$\begin{array}{r} \frac{dI\left(t\right)}{dt}=\gamma E\left(t\right)-\delta I\left(t\right) \end{array}$$

(9) $$\begin{array}{r} \frac{dQ\left(t\right)}{dt}=\delta I\left(t\right)-\lambda \left(t\right)Q\left(t\right)-\kappa \left(t\right)Q\left(t\right) \end{array}$$

(10) $$\begin{array}{r} \frac{dR\left(t\right)}{dt}=\lambda \left(t\right)Q\left(t\right) \end{array}$$

(11) $$\begin{array}{r} \frac{dD\left(t\right)}{dt}=\kappa \left(t\right)Q\;\left(t\right) \end{array}$$

In the traditional epidemiological model, the parameters are determined by natural history of the disease. In the GPCP system, the parameters of the model are obtained from the actual epidemic data inversion. The dynamics of each population group are governed by the parameters β, γ, λ, and κ (unit: day^−1^). β is the infection rate, which represents the average number of susceptible persons in effective contact with each sick person per day. 1/γ is the average latent time (the time between getting infected and onset of symptoms). λ and κ are the recovery rate and death rate, respectively. α is the parameter of social distancing. When α > 0, individuals are passed from group S to group P, indicating the implementation of social distancing measures. The infectious will then spread the virus to others before admitted to the hospital at the rate of δ (entering the quarantined stage, Q). The influence of temperature and humidity has been included in the improved SEIR [see Huang et al. (18) for details], and the influence of mass gatherings on the epidemic situation is mainly considered here. The number of asymptomatic infections was calculated considering the number of mass gatherings. About 15.6% people are asymptomatic infections among confirmed cases (95% CI, 10.1–23.0%) (19). Therefore, we calculated the number of asymptomatic infections among mass gatherings using the following formula:

(12) $$\begin{array}{r} E_{a}\left(i\right)=0.156\times Pot\left(i\right)\times Con\left(i\right)÷Npop \end{array}$$

where Pot(i) is the daily number of flood victims reported by the National Disaster Management Authority Government of India for the 2020 summer outbreak (hereinafter referred to as the first outbreak). For 2021 spring outbreak (hereinafter referred to as the second outbreak) in India, Pot(i) is the number of religious congregations reported by news published online. Con(i) is the number of cumulative confirmed cases on day I, and Npop is the population of India.

## Results

### The Timeline of Development of Global COVID-19

**Figure 1:**
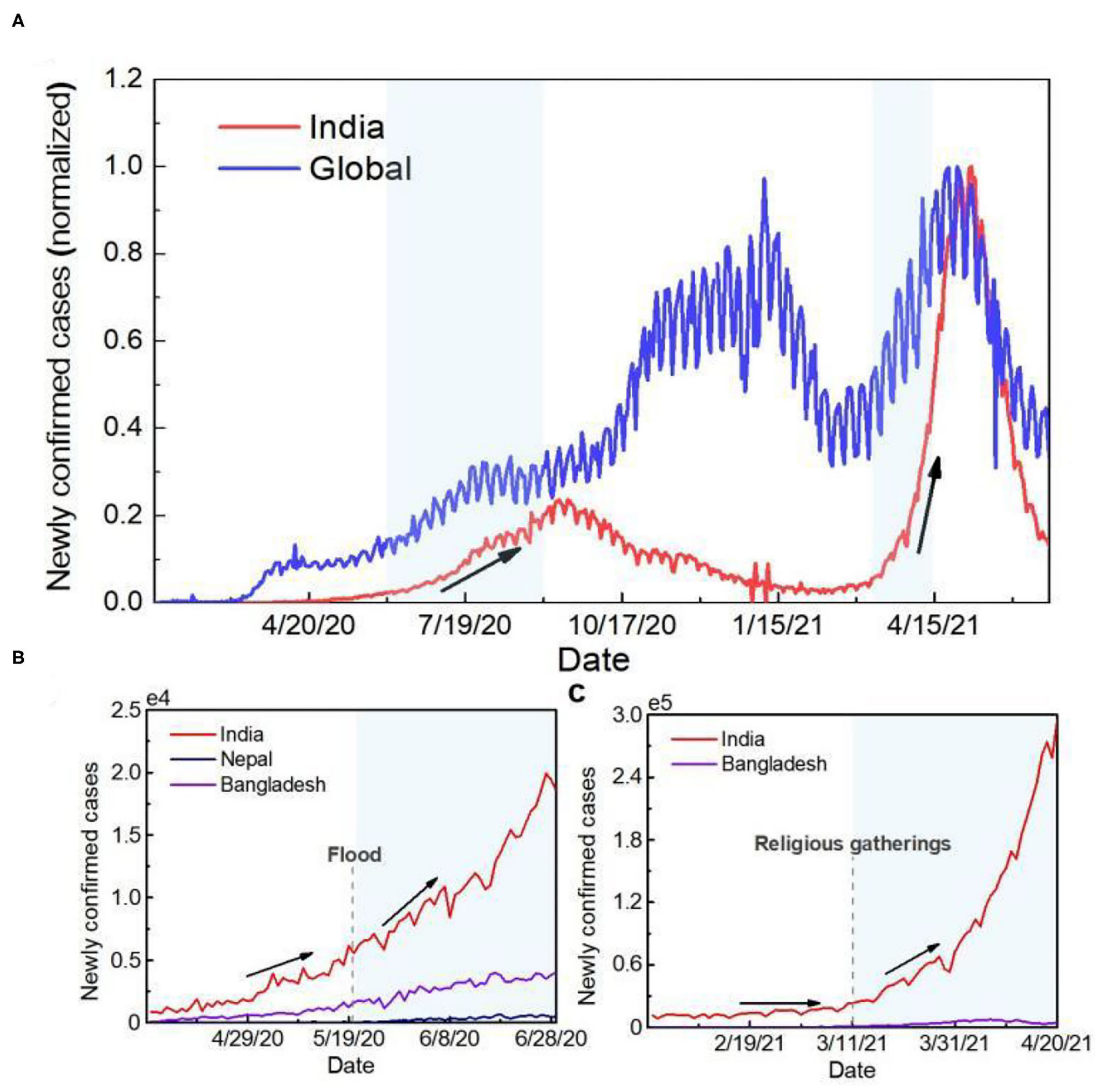
Time series of the number of newly confirmed cases. **(A)** Time series curve of newly confirmed cases globally and in India. To facilitate the comparative analysis, the data were normalized. The red line represents newly confirmed cases in India, and the blue line represents global new cases. **(B)** Time series curve of newly confirmed cases in countries mainly affected by flooding during the first outbreak in India. The dotted line represents when the flood started. **(C)** Time series curve of newly confirmed cases in India and Bangladesh during the second outbreak in India. The dotted line represents the time when the crowd gathered.

According to previous studies, the COVID-19 pandemic was characterized by oscillatory outbreak patterns, with anthropogenic factors, natural disaster-related factors, and seasonal temperature changes as triggers (18). Therefore, we attempt to find possible trigger factors of the outbreaks in India based on the timeline of development of COVID-19 (Figure 1). Two COVID-19 waves have affected India since its first patient was identified on 30 January 2020 (Figure 1A). India experienced the first (2020 summer) outbreak when the global situation was relatively stable. After March 2021, when the trend of the global epidemic was declining, India had another outbreak, with the daily new confirmed cases peaked at 57.1% of the global total.

Based on the timing, the potential trigger for the first outbreak in India could be the effects of a natural disaster. As of 1st June 2020, the number of cumulative confirmed cases in India was <200,000, but more than 3 million new cases were reported within 3 months after the start of the monsoon season. Since the super-cyclone “Amphan,” the number of confirmed cases in India, Bangladesh and Nepal also increased by 335.5, 337.3 and 2967.2%, respectively, in 40 days (Figure 1B). The second outbreak in India was caused by human factors. Since 11th March 2021, nearly 3,000,000 people have bathed in the Ganges River for Kumbh Mela, providing an ideal situation for the transmission of the virus. After Kumbh Mela, the number of confirmed cases in India increased by 665.5% within 1 month. Meanwhile, the time series of COVID-19 pandemic development in Bangladesh, downstream of India, was consistent with that in India. The number of daily new cases was over two times higher than the previous highest record (Figure 1C).

### Causes of the 2020 Summer Outbreak in India

**Figure 2:**
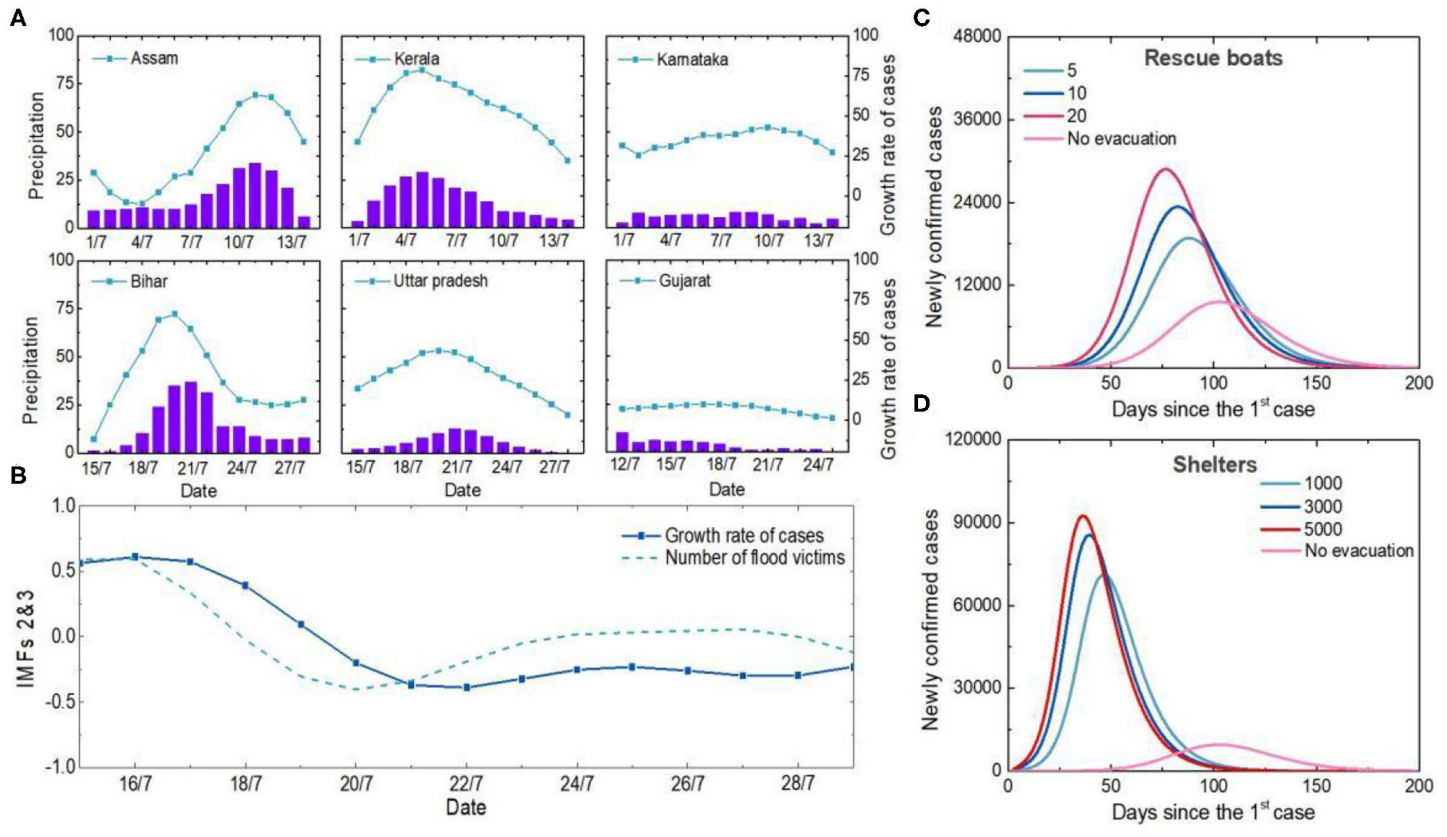
Analysis of the causes of the first outbreak in India. **(A)** Temporal variations in precipitation and newly confirmed cases in Indian states. The purple column represents precipitation, and the blue symbol line represents the 5-day increase in the number of newly confirmed cases. **(B)** Relationship between number of flood victims and newly confirmed cases. The second and third components of the EEMD were extracted as detrending items, and all data were normalized. **(C)** Influences of different boat carrying capacities on the number of cases. Curves in different colors represent the simulation results of newly confirmed cases under different carrying numbers of each rescue boats. **(D)** Impacts of different shelter populations on the epidemic. The curves in different colors represent the simulation results of newly confirmed cases with different numbers of shelters.

The cause of the first outbreak in India was further traced systematically (Figure 2). During the first outbreak, the numbers of COVID-19 confirmed cases are closely related to monsoon season precipitation. In Assam, Bihar, Kerala and Uttar Pradesh, the correlation coefficients between precipitation and the number of confirmed cases during floods were 0.87, 0.84, 0.95 and 0.87, respectively (Figure 2A). There is a lag correlation between the increases of confirmed cases and the number of flood victims (correlation coefficient = 0.87) (Figure 2B). During flood, while rescue procedures or refugee camping, it is almost impossible to follow COVID-19 protocols, so protective mask and social distancing is limited. Population densities and intensity of social contacts are the main drivers for propagation and amplification of this novel respiratory virus SARS-CoV-2 (20). In addition, as environmental changes occur, vector breeding sites increase, and access to healthcare services is limited, etc, the after-effects of flooding may contribute to the occurrence and the spread of infectious diseases (21) (Supplementary Figure S1).

Unplanned and overcrowded shelters, limited rescue personnel and equipment all contribute to large crowd gatherings and facilitate the spread of viruses. Simulation results show that more than 5, 10 and 20 people on each boat without personal protection would increase the number of new confirmed cases by 68.2, 97.1, and 129.6%, respectively (Figure 2C). Shelters housing 3,000 victims would lead to a four-fold increase in the peak number of new confirmed cases if anti-epidemic measures were not taken (Figure 2D). Therefore, human-to-human transmission among dense populations during natural disaster events was the main reason why India was one of the most COVID-19-affected countries in the summer of 2020.

### Causes of the 2021 Spring Outbreak in India

**Figure 3:**
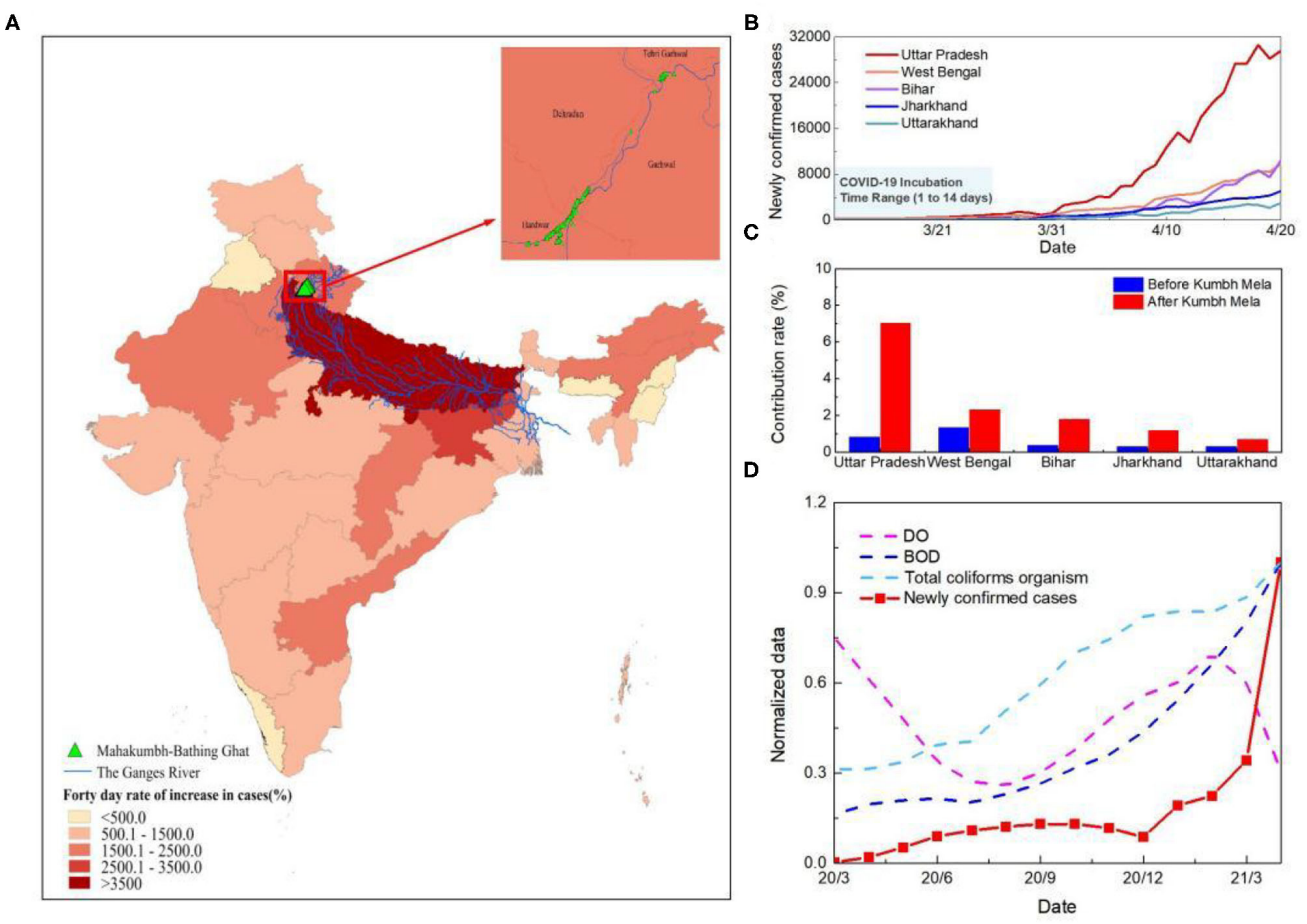
Analysis of the causes of the second outbreak in India. **(A)** Spatial distribution of the growth rate of the number of cases at 40 days since Kumbh Mela in India. **(B)** Time series curve of newly confirmed cases in five states along the Ganges River basin in India. The light blue shaded area represents the incubation period of SARS-CoV-2. **(C)** Change in the number of new cases as a percentage of India's total in the five states in the Ganges basin. The columns represents the proportion of newly confirmed cases at 40 days before and after Kumbh Mela. Blue is pre-Kumbh Mela and red is post-Kumbh Mela. **(D)** Time series of water quality and new confirmed cases in Uttar Pradesh. The red line represents the number of new confirmed cases and the other three dotted lines represent DO, BOD, and Total coliforms organism. To facilitate the comparative analysis, the data were normalized.

Close physical contact facilitates easy and rapid spread of respiratory pathogens at gatherings. Upper respiratory tract infection (URTI) has been reported as the main cause of illness among Kumbh Mela pilgrims in 2013, accounting for 70% of the illness among the pilgrims (22). However, the super outbreak in India in 2021 spring was probably not only caused by the high density of human-to-human transmission. While COVID-19 cases have increased significantly in all Indian states since the start of Kumbh Mela, the increase has been even more rapid in the Ganges Basin (Figure 3A). The percentage of the number of new cases in the states along the Ganges River basin to the total number of new cases in India increased by about threefold within 40 days (Figures 3B,C). This is not significantly related to the movement of people among the states during Kumbh Mela (Supplementary Figure S2). In Uttar Pradesh, the state closest to the holy bathing sites, the proportion has increased six-fold than that before Kumbh Mela. Cities that run through the Ganges contributed 92.5% of newly confirmed cases in the state, including the five cities with the worst outbreaks (account for 48.8%) (Supplementary Figure S3).

Previous studies on the impact of sewage discharge on groundwater pollution have found e. coli in groundwater samples exceeding the permissible limit of WHO, forcing the belief that if it can contaminate and present in a significant amount, then concerning COVID can also penetrate to groundwater (23). Therefore, to further trace the reasons for the rapid development of the epidemic in the Ganges basin, a comparative assessment of dissolved oxygen (DO), biochemical oxygen demand (BOD) and total coliforms organism of river Ganga in Uttar Pradesh was conducted (Figure 3D). The results demonstrate an increase of microbiological contamination of surface water during the COVID-19 outbreak. The average value of BOD and total coliforms organism increased from 3.8 mg/L to 4.5 mg/L and 17652.3 MPN/100 ml to 18767.6 MPN/100 ml, respectively, while the average DO decrease from 8.7 to 7.7 mg/L, indicating the higher load of organic pollution in the river system during Kumbh Mela. The slight decrease in DO observed during the two outbreaks which may also be due to the increased levels of suspended solids and turbidity in the river water because of heavy rains and holy bathing (24).

The increase of microbiological contamination of surface water is likely to warn of future biological pandemics, including COVID-19. Studies have reported that SARS-CoV-2 RNA in an Italian and a Japanese river (25, 26). Sewage samples from Gujarat state, India also showed presence of SARS-CoV-2 RNA (27). If SARS-CoV-2 RNA is detected on water or sewage, infectivity cannot be ruled out (28). The emergence of human pathogenic viruses in aquatic ecosystems needs to raise concerns about environmental and human health-related impacts.

### Simulation of the Two Outbreaks in India

**Figure 4:**
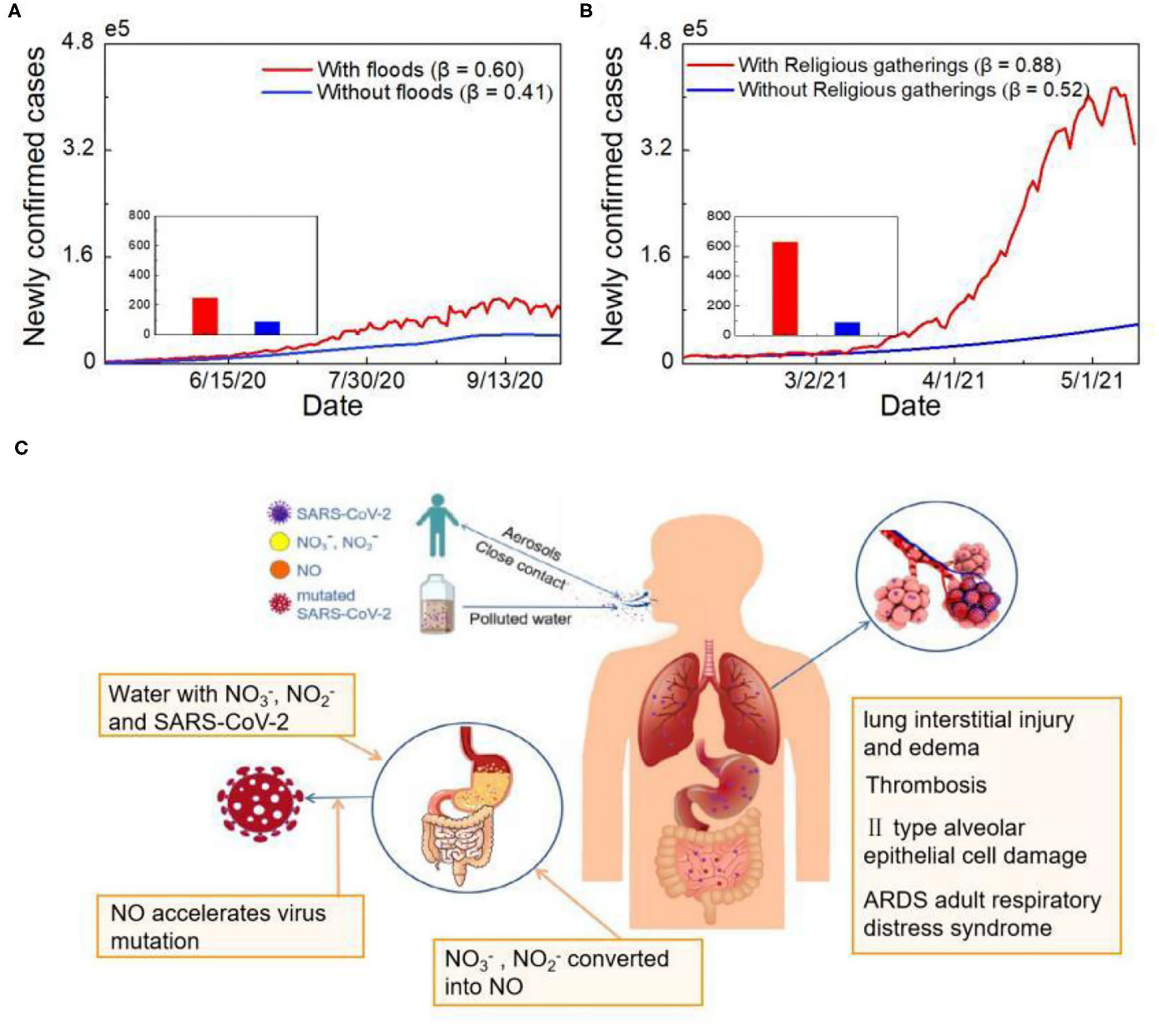
Simulated analysis of COVID-19 human-to-human and water-to-human transmission. **(A)** Epidemic simulation of newly confirmed cases without floods. The red line represents the actual number of cases, and the blue line shows the number of newly confirmed cases simulated without floods. **(B)** Epidemic simulation of newly confirmed cases without holding Kumbh Mela. The red line represents the actual number of cases, and the blue line shows the number of newly confirmed cases simulated without religious gatherings. **(C)** The COVID-19 human-to-human and water-to-human transmission processes.

According to the simulation results, India should not have experienced such a severe outbreak. Flood-triggered high-density human-to-human transmission during the first outbreak doubled the number of COVID-19 cases in India (Figure 4A, Supplementary Figure S4). The combination of human-to-human and water-to-human transmission during the second outbreak resulted in a six-fold increase in newly confirmed cases (Figure 4B). Human-to-human transmission increased Uttar Pradesh's share of the total number of cases in India by 0.5%. After adding to the impact of water-to-human transmission, the proportion increased by 6.3% (Supplementary Figure S5). Same size of Massive gathering event (MGE) in India has led to a three-time increase rate of the confirmed cases compared to the US (18). Therefore, it is quite possible that water-to-human transmission amplified the epidemic and caused the most severe outbreak worldwide.

The course of SARS-CoV-2 infection in pulmonary and gastrointestinal (GI) tissues is presented in Figure 4C. A large number of viral-containing particles can be inhaled by people who are exposed to a high number of human-generated aerosols for a long duration; thus, the chance of becoming infected and the disease severity is high (29). Greater inhalation expands the alveolar area exposed to virus-containing aerosols, which may affect viral processing and the immune response (30). Similar to fecal-oral transmission, SARS-CoV-2 can enter human body directly through water-oral transmission route. Previous research has shown that GI symptoms affect up to 26% of patients in some populations, with some patients experiencing only digestive symptoms (31). The virus-specific RNA and protein synthesis processes can form virions in the cytoplasm and then release them to the GI tract. Moreover, other contaminants in water may promote virus mutation (32). Based on previous research, the nitrate and nitrite levels in the Ganges River transect exceed the standard level (33). Excess nitrate and nitrite are further converted into nitric oxide with the help of oral bacteria, enzymes and acid in the stomach (34). Oxidative stress induced by the overproduction of nitric oxide can trigger virus mutation; for example, it can cause heterogeneity among variants of RNA viruses, such as HIV and influenza, and accelerate viral evolution (Figure 4C).

## Discussion

The presence of multiple transmission mechanisms for COVID-19 is one of the reasons for the rapid spread of the pandemic. Fecal-oral transmission as a potential transmission mechanism has attracted a lot of attention in recent years, but there is still a lack of epidemiological evidence to confirm this hypothesis. Replication of the virus in the intestinal tract is highly likely (35). The unsuccessful isolation of infective SARS-CoV-2 from stool and wastewater samples may be due to the difficulty of isolating intact enveloped virions, rather than the absence of infective virions (36). During the March 2003 outbreak of SARS in Hong Kong, evidence of fecal-aerosol transmission route was reported due to aerosol diffusion from inadequate wastewater management (37). Thus, wastewater should be alert to contain a considerable number of infective virions. To date, there is no clear evidence regarding SARS-CoV-2 survival in water or sewage. However, due to the presence of virus fragments in excreta, as well as other potential infectious disease risks in excreta, wastewater should be treated in a well-designed and well-managed central sewage treatment plant (38).

COVID-19 water-to-human transmission route is a warning of water security issues during an epidemic. Especially the biological treatment of medical wastewater and domestic sewage. To reduce the risk of river-borne infection, it is necessary to strengthen the management of water supply and drainage systems, and formulate reasonable policies to limit the flow of people along the river. In countries with highly developed water supply systems, it is difficult for the virus to overcome the existing stages of filtration and disinfection. In contrast, the presence of the virus is unknown in countries where water treatment technology does not have the equipment to remove it (39). According to the United Nations' 2017 World Water Development Report, 80% of global wastewater (>95% in some developing countries) is released to the environment without adequate treatment (40). However, in the context of the COVID-19 pandemic, there is little information on the presence of SARS-CoV-2 in sewage and natural waters in countries with poor sanitation. Extensive research on the detection of infectious SARS-CoV-2 in wastewater is urgently needed. A large population in India is affected by water security issues and climate-induced disasters, such as floods and droughts (41, 42). Transient populations, urbanization and traditional and contemporary recreational activities result in a high pollution load and increase future environmental risk. Therefore, it is essential to deepen the understanding of the effect of ecological safety and human health and to develop corresponding risk management and environmental modification plans, especially during the high-incidence infectious disease season. Currently, wastewater epidemiology should be brought to the forefront of disease research. However, low coverage (one-third of all towns) of sewer networks in India makes conducting such research challenging.

This study provides a new perspective that virus mutations are largely related to human activities. This indicates that strict social distancing can not only break the transmission chain and prevent infection but also prevent virus mutation. Global mass infection, vaccination, and interventions are changing SARS-CoV-2's evolutionary landscape. Since the first outbreak period in India, effective global medical interventions have been developed. However, the vaccination coverage rate is still too low to allow pandemic control (43, 44). At the same time, viruses keep evolving to become more infectious and overcome a host's immune responses (45). The omicron variant, for example, is currently causing a global super-outbreak, evades the immune protection offered by vaccines and natural infections (46). Therefore, strict non-pharmaceutical interventions should remain the first line of defense against the virus.

In addition, based on the results of the attribution analysis of the first outbreak in India, we model that without rigorous interventions, the cumulative number of cases in India's 2021 monsoon season could reach 50 million by the end of July, resulting in many lives lost (Supplementary Figure S6). It is a substantial challenge to the society, requiring relevant departments to make complex, highly compromised, hierarchical decisions to address the pandemic. Accelerating vaccination, expanding viral genome testing and implementing strict distance restrictions to prevent transmission and save lives should be regarded as priorities.

## Data Availability Statement

The original contributions presented in the study are included in the article/Supplementary material, further inquiries can be directed to the corresponding author/s.

## Author Contributions

JH designed the study and contributed to the ideas, interpretation, and manuscript writing. XL contributed to interpretation and manuscript writing. All authors contributed to the discussion, interpretation, and reviewed the manuscript. All authors contributed to the article and approved the submitted version.

## Funding

This work was jointly supported by the National Natural Science Foundation of China (41521004) and the Gansu Provincial Special Fund Project for Guiding Scientific and Technological Innovation and Development (Grant No. 2019ZX-06).

## Conflict of Interest

The authors declare that the research was conducted in the absence of any commercial or financial relationships that could be construed as a potential conflict of interest.

## Publisher's Note

All claims expressed in this article are solely those of the authors and do not necessarily represent those of their affiliated organizations, or those of the publisher, the editors and the reviewers. Any product that may be evaluated in this article, or claim that may be made by its manufacturer, is not guaranteed or endorsed by the publisher.
