# Supplementary material for: Water Transmission Increases the Intensity of COVID-19 Outbreaks
Source: Front Public Health. 2022 May 25;10:808523. doi: 10.3389/fpubh.2022.808523 (PMC9174688; doi:10.3389/fpubh.2022.808523)
Supplement: Supplementary file 1 [file Data_Sheet_1.pdf]

Supporting Information for

Water transmission increases the intensity of COVID-19 outbreaks

J. Huang[1]*, X. Lian[1] , Y. Zhao[1], D. Wang [1], S. Chen[1]
L. Zhang [1], X. Liu[1] , J. Gao[1] and C. Liu[1]

[1]Collaborative Innovation Center for West Ecological Safety (CIWES), College of Atmospheric Sciences, Lanzhou University, Lanzhou, 730000, China.

Corresponding author: Jianping Huang (hjp@lzu.edu.cn)

Contents of this file

Fig. S1-S7

Introduction

This supporting information consists of seven figures.

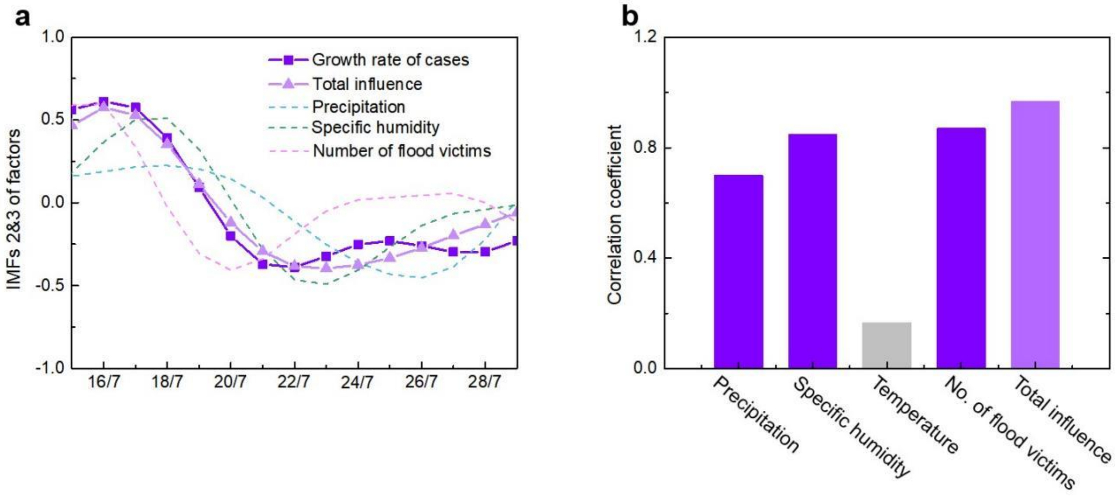

Figure. S1. Potential effects of different factors on COVID-19 transmission. (**a**) Time series of different factors. Total influence represents the sum of the three terms of precipitation, specific humidity, and the number of flood victims. The second and third components of EEMD were extracted as de-trending items, and all data was normalized. (**b**) Correlation analysis of different influencing factors and confirmed cases.

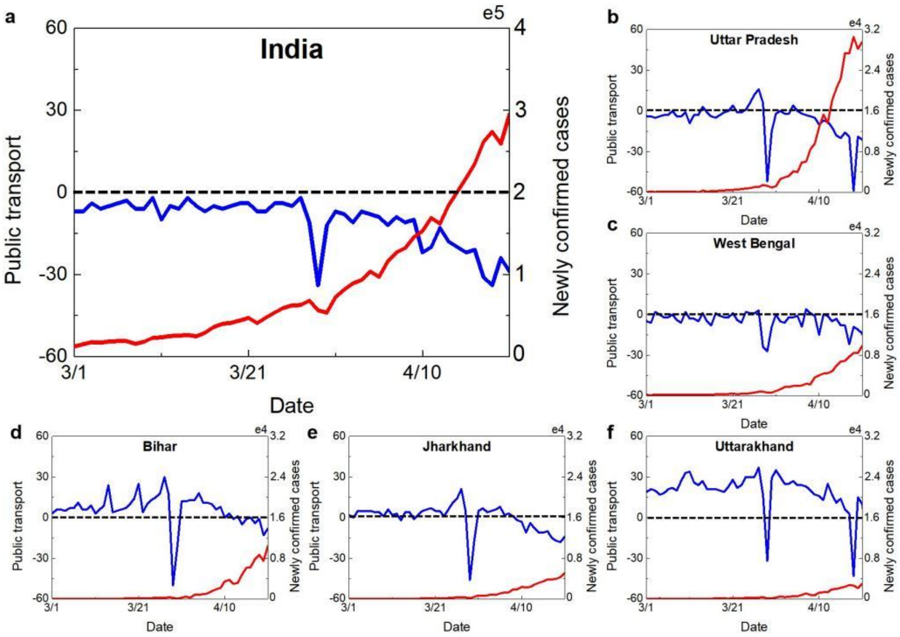

Figure. S2. The impact of public transport flows on India's second outbreak. (**a**) Time series curve of public transport and the number of new confirmed cases in India. The blue line represents mobility trends for places that are public transport hubs, such as underground, bus and train stations. Changes for each day are compared to a baseline value (black dotted line) for that day of the week. The data is available at google.com/covid19/mobility. The red line is the number of new confirmed cases. (**b**)-(**f**) Time series curve of public transport and the number of new confirmed cases in five states along the Ganges River basin in India.

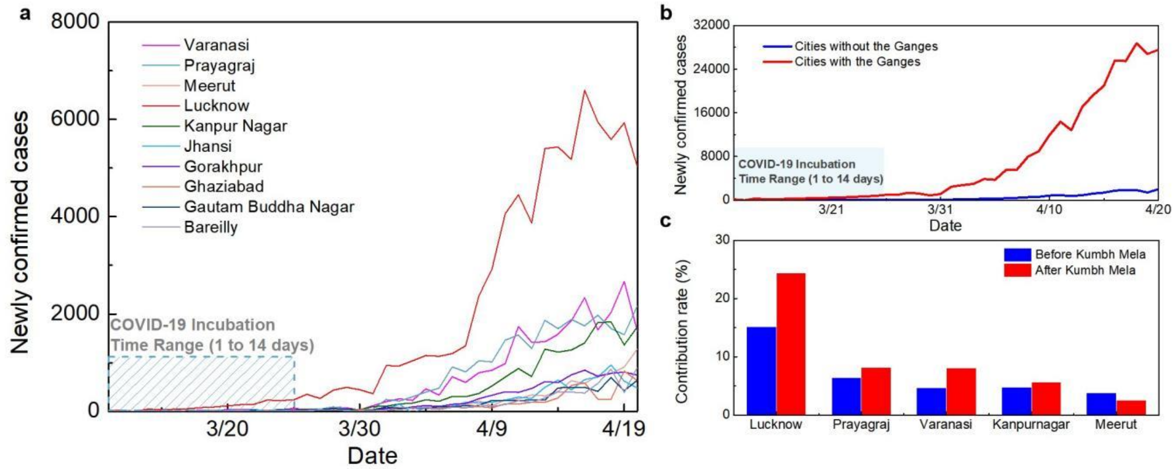

Figure. S3. The epidemic time series curve of cities in Uttar Pradesh. (**a**) The epidemic time series curve of the top 10 cities with COVID-19 cases in Uttar Pradesh. Basically, the total number of cases in the top five cities in Uttar Pradesh can representative of the outbreak in the state. (**b**) Time series curve of newly confirmed cases in areas contacting and not contacting the Ganges River. (**c**) Change in the number of new cases as a percentage of Uttar Pradesh's total in the top five cities.

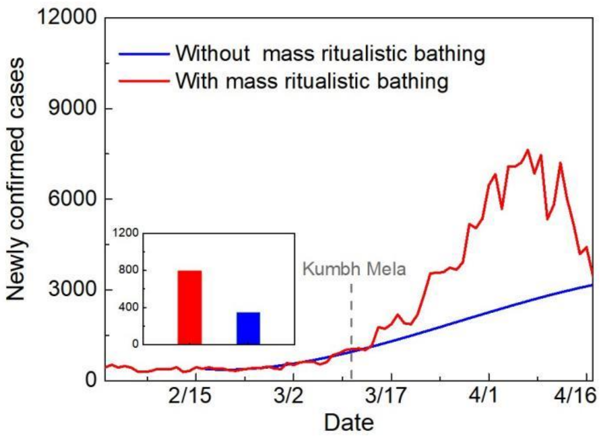

Figure. S4. Epidemic simulation of new confirmed cases without Kumbh Mela in Bangladesh. The blue line is the number of new confirmed cases without religious gatherings. The red line represents the actual data reported. The red column represents the growth rate of new confirmed cases in the 40 days after Kumbh Mela, and the blue column represents it without Kumbh Mela.

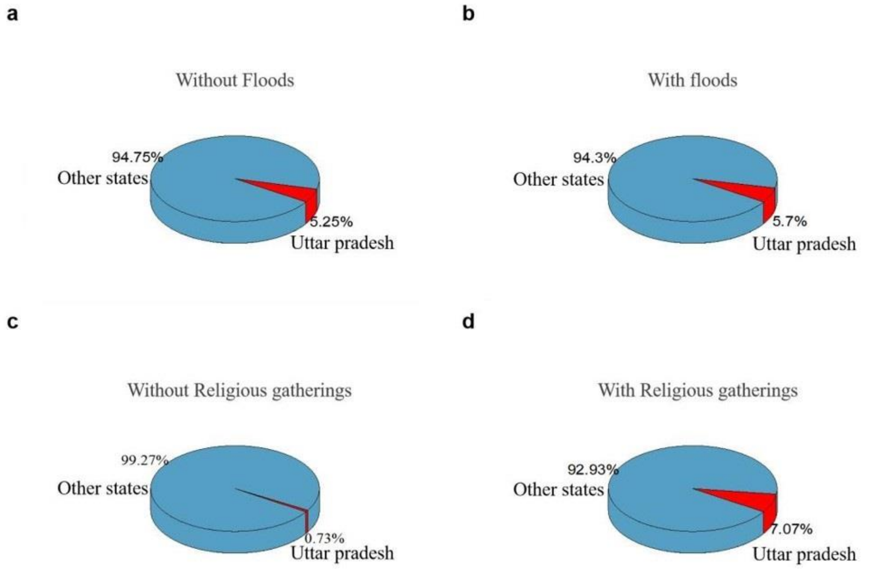

Figure. S5. Uttar Pradesh's contribution rate to COVID-19 cases under different scenarios. (**a**) and (**b**) The proportion of Uttar Pradesh's COVID-19 cases in the total number of cases in India with and without floods. The red represents Uttar Pradesh and the blue represents the proportion of other Indian states. (**c**) and (**d**) The proportion of Uttar Pradesh's COVID-19 cases in the total number of cases in India with and without Kumbh Mela. The red represents Uttar Pradesh and the blue represents the proportion of other Indian states.

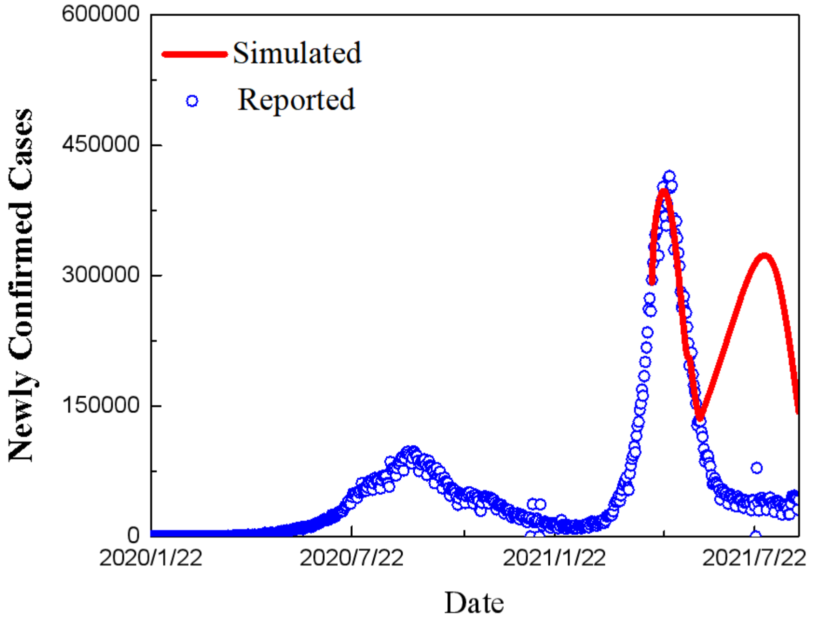

Figure. S6. Simulation of the COVID-19 epidemic in India without strict interventions in Monsoon Season. The red line represents the simulation, and the blue circle represents the actual number of cases reported.

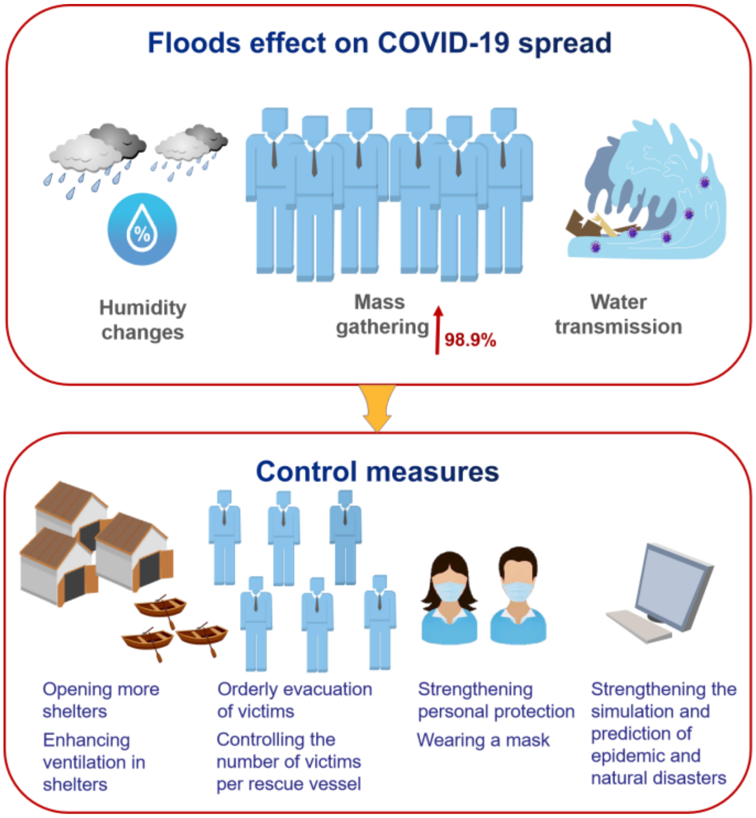

Figure. S7. Control scheme fighting against the dual-disaster of COVID-19 and flooding during the monsoon season
